# Designing a model of emergency medical services preparedness in response to mass casualty incidents: a mixed-method study

**DOI:** 10.1186/s12873-024-01055-1

**Published:** 2024-07-24

**Authors:** Vahid Saadatmand, Milad Ahmadi Marzaleh, Nasrin Shokrpour, Hamid Reza Abbasi, Mahmoud Reza Peyravi

**Affiliations:** 1 Department of Medical Emergencies, School of Paramedicine, Faculty of Jahrom University of Medical Science, Jahrom, Iran; 2 Department of Health in Disasters and Emergencies, School of Health Management and Medical Information Sciences, Faculty of Shiraz University of Medical Sciences, Shiraz, Iran; 3 English Department, Faculty of Paramedical Sciences, Shiraz, Iran; 4 Department of Surgery, School of Medicine, Faculty of Shiraz University of Medical Sciences, Shiraz, Iran; 5 Department of Health in Disasters and Emergencies, School of Health Management and Medical Information Sciences, Faculty of Shiraz University of Medical Sciences, Alley 29, Qasrodasht Ave, Shiraz, 71336-54361 Iran

**Keywords:** Disaster, Mass casualty incident, Emergency medical service, Preparedness, Model

## Abstract

**Background:**

Emergency medical services preparedness in mass casualty incidents is one of the most important concerns in emergency systems. A mass casualty incident is a sudden event with several injured individuals that overwhelms the local health care system. This study aimed to identify and validate the components of emergency medical services readiness in mass casualty incidents which ultimately led to designing a conceptual model.

**Methods:**

This research was an explanatory mixed-method study conducted in five consecutive stages in Iran between November 2021 and September 2023. First, a systematic review was carried out to extract the components of emergency medical services preparedness in mass casualty incidents based on the PRISMA guideline. Second, a qualitative study was designed to explore the preparedness components through in-depth semi-structured interviews and analyzed using the content analysis approach. Third, the integration of the components extracted from the two stages of the systematic review and qualitative study was done by an expert panel. Fourth, the obtained components were validated using the Delphi technique. Two rounds were done in the Delphi phase. Finally, the conceptual model of emergency medical services preparedness in mass casualty incidents was designed by a panel of experts.

**Results:**

10 articles were included in the systematic review stage and sixteen main components were extracted and classified into four categories. In the second stage, thirteen components were extracted from the qualitative study and classified into five categories. Then, the components of the previous two phases were integrated into the panel of experts and 23 components were identified. After validation with the Delphi technique, 22 components were extracted. Lastly, the final components were examined by the panel of experts, and the conceptual schematic of the model was drawn.

**Conclusions:**

It is necessary to have an integrated framework and model of emergency medical service readiness in the planning and management of mass casualty incidents. The components and the final model of this research were obtained after the systematic scientific steps, which can be used as a scheme to improve emergency medical service preparedness in response to mass casualty incidents.

## Background

Mass casualty incidents (MCIs) are among the critical issues that affect the healthcare system including emergency medical service (EMS). The World Health Organization (WHO) defines MCIs as disasters characterized by quantity, severity, and variety of patients that can immediately overwhelm the capability of local medical resources to deliver comprehensive medical care. MCIs occur when the demand for medical services brought on by a sudden event exceeds the capacity of a healthcare system to supply them [1, 2]. MCIs span multiple causes including natural disasters, transport incidents, terrorism, armed conflicts, etc. MCIs are often devastating to societies and have profound mortality and long-term consequences [3]. EMS is a part of the health system that is assigned to emergency response. The WHO considers EMS systems as an integral part of any operational healthcare system. EMS provides care to people in a variety of medical emergencies. One of the most important responsibilities of EMS is to respond to MCIs. When an EMS response involves large casualties, responsible organizations such as the police and fire and rescue teams must utilize resources to expand operations. In large MCIs, there may be more victims than regional EMS can manage, and additional assistance may be required. When the resources are limited or the EMS is involved in the number of casualties, the disaster happens. In most MCIs, routine operations may be developed to manage increased capacity. In the United States, MCIs are managed using the National Incident Management System (NIMS) and the Incident Command System (ICS). All rescue providers and disaster management workforce should be familiar with and competent in using NIMS and ICS [4, 5].

EMS plays a major role in managing all phases of MCIs, including prevention, preparedness, response, and recovery [6]. These systems require an organized and planned approach with adequate resources so that they can effectively manage MCIs. Such important tasks of this system are life-saving measures, triage, and transfer of the injured to medical centers. The EMS system should increase its level of preparedness in response to such disasters. This requires planning, training, coordinating, and sensitizing managers to pay more attention to this area [7]. Therefore, EMS systems must design a framework based on standard indices. To this end, this study was conducted to identify the components of EMS readiness in MCIs and design a model that can depict the dimensions of EMS preparedness in response to MCIs.

### Conceptual framework

The conceptual framework of this research in line with the study objectives includes three components: emergency medical service (EMS), preparedness, and mass casualty incidents (MCIs).

### Conceptual view of emergency medical service (EMS)

An Emergency Medical Service can be defined as "a comprehensive system which provides the organization of staff, facilities, and equipment for the effective, coordinated, and timely delivery of care and emergency services to victims of sudden illness or injury." The ambition of EMS focuses on providing timely care to the victims of sudden and life-threatening injuries or emergencies to prevent undesired mortality or long-term morbidity. The function of EMS can be simplified into four main components: accessing emergency care, care in the community, care en route, and care upon arrival to receiving care at the healthcare facility [8, 9]. In addition to the routine responsibility of EMS, one of the important tasks of this system is disaster management [2].

### Conceptual view of preparedness

Preparedness is the knowledge and capacities developed by governments, professional response and relief organizations, communities, and individuals to effectively anticipate, respond to, and recover from the impacts of likely, imminent, or current hazards, events, or situations [10]. Preparedness is a continuous cycle of planning, organizing, training, equipping, exercising, evaluating, and taking corrective action to ensure effective coordination during the incident response [11]. Preparedness within the field of emergency management can best be defined as a state of readiness to respond to a disaster, crisis, or any other type of emergency [12, 13]

### Conceptual view of mass casualty incidents (MCIs)

A Mass Casualty Incident (MCI) can be defined as "an overwhelming event, which generates more victims at a time than locally available resources can manage using routine procedures. It requires exceptional emergency arrangements and additional or extraordinary assistance." MCIs can occur as a consequence of a wide variety of events: disasters (both natural and man-made), terrorist attacks, motor vehicle collisions, etc. Whatever the cause of the event is, the characterizing feature of an MCI is the number of victims large enough to disrupt the normal functioning of healthcare services. MCIs can be classified into different levels according to either the number of potential victims or the entity of the response [14]**.**

## Methods

This research was an explanatory mixed method suggested by Creswell [15] which was conducted in five consecutive stages in Iran between November 2021 and September 2023. The purpose of this mixed-method study was to identify the main components of EMS preparedness in MCIs in several systematic stages and then design a conceptual model that can represent the essential elements of EMS readiness in MCIs in the form of a framework. The questions of this research were as follows:

What are the main and effective components of EMS preparedness in response to MCIs?

What will be the final model of EMS readiness in response to MCIs?

**Fig. 1:**
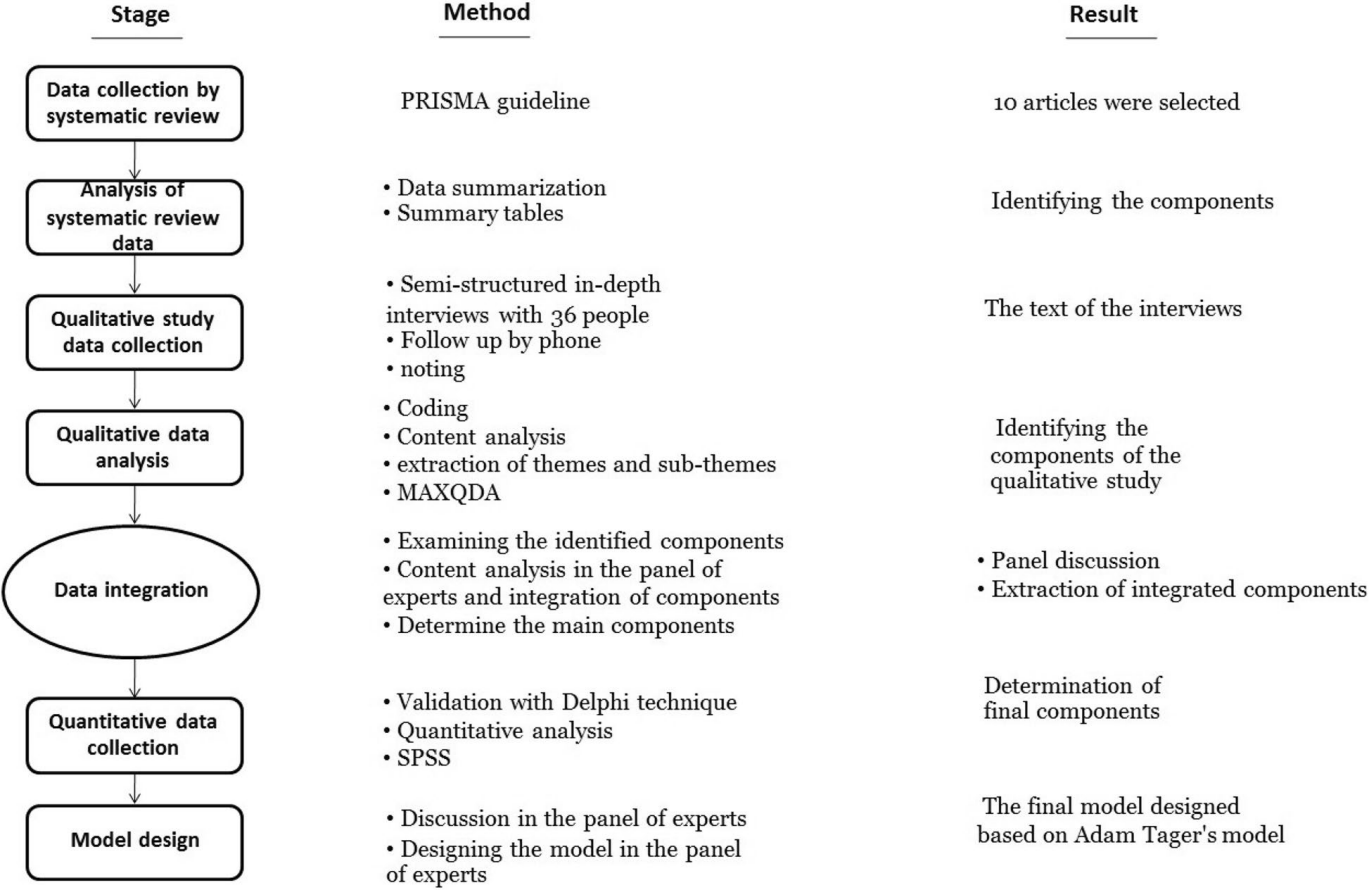
Research implementation diagram

The research implementation diagram is shown in Fig. 1.

## Systematic review

### Eligibility criteria and search strategies

This study was conducted based on the Preferred Reporting Item for Systematic Reviews and Meta-analyses (PRISMA-ScR) instruction. The systematic review instruction included the background data, review questions, inclusion and exclusion criteria, search strategies, selection of studies, criteria to review the studies, literature review, data extraction, and reporting. A systematic search was performed from January 1970 to February 2022 in peer-reviewed English texts related to the research question—i.e., what the components of EMS preparedness for MCIs are. First, a rapid and general search was conducted in the Cochrane Library database to ensure the lack of duplicate studies. The results indicated that there were no duplicate articles. The electronic databases searched included PubMed, Cochrane Library, Scopus, Science Direct, and ProQuest. The gray literature, such as books, theses, conference articles, and websites, was also searched. The “AND” operator was used to search among the groups of words considered as separate concepts. For synonym words, the “OR” operator was used. The search was conducted on the titles, abstracts, and keywords of the articles. Medical Subject Headings (MeSH) terms were used to find articles in the PubMed database. The search strategy is presented in Table 1.

**Table 1:** The search strategy employed to determine the components of EMS preparedness in MCIs

PIO	#1 AND #2 AND #3	Strategy
P	"Emergency Medical Services" OR "Prehospital" OR "Paramedic" OR "Prehospital Emergency Care" OR " Emergency Medical Technician"	#1
I	"Catastrophe" OR "Disaster" OR "Emergency" OR "Accident" OR "Incident simulator " OR "Event" OR "Mass Casualty Incident" OR "Multiple Casualty Incident" OR "Mass Destruction" OR "Mass Destruction of Weapon" OR "Terrorist" OR "Chemical, Biologic, Radioactive, Nuclear, Explosive"	#2
O	"Preparedness" OR "Preparation" OR "Readiness" OR "Response"	#3

The keywords were selected using MeSH and investigating the studies based on the objectives. In the next step, a full list of references for all the articles was prepared, and the titles of the articles were investigated by the researchers to omit the irrelevant ones. The END NOTE software, version X8, was used to manage the resources.

### Inclusion criteria

The main keywords were used to explore the studies related to EMS preparedness in MCIs. First, the titles of the articles were evaluated by two independent reviewers. We determined whether titles, keywords, or abstracts were included in the study subject. In the case of disagreement, they were verified by one external reviewer using the consensus method, and any disagreement was resolved. Then, the abstracts were assessed; finally, the full texts of the articles were evaluated based on the validated checklists. The STROBE (Strengthening the Reporting of Observational Studies in Epidemiology) and the COREQ (Consolidated criteria for reporting qualitative research) checklists were used for the quality evaluation of the studies. The included articles were written in the English language. Unpublished articles (gray literature), protocols, conference papers, guidelines, and reports from creditable organizations were reviewed as well. To analyze the risk of bias, two researchers independently performed all quality assessment processes based on the Cochrane Collaboration tools.

### Exclusion criteria

The articles in the field of hospital emergency department, those irrelevant to the research questions, guidelines outside the scope of the study, and the articles that did not meet the expected quality according to the validated checklists were excluded from this study.

### Screening

First, the titles of all the articles extracted from the databases were checked by two independent reviewers. The articles that met the inclusion criteria and were relevant to the research question were selected. In the next step, the author studied the abstracts of the selected articles. Then, those abstracts that were completely in line with the study objectives and the inclusion criteria were extracted, and their full texts were assessed by the authors. Finally, the articles relevant to EMS preparedness in MCIs were selected.

### Data extraction

The essential information was extracted after reading the articles carefully based on the summaries and collection forms. This form included the title, corresponding author, research objective, research population, samples, country, time of research, research design, instruments, method, results, and conclusion sections. The summary forms were filled out for each article. These forms were evaluated by two independent researchers after reviewing all the articles; the data were then presented in a table. If there were disagreements in data extraction, an external researcher commented on the conflicting issues, and any disagreement was resolved [16].

## Qualitative study

### Design

This qualitative study was conducted using a content analysis approach. Inductive coding was adopted in this approach. Recently, three approaches have been presented in content analysis, including conventional, summative, and directed, which differ in coding and trustworthiness. In the conventional approach, classification codes are extracted directly from the data [17]. The study adopted the conventional content analysis as well. This study was conducted using the consolidated criteria for reporting qualitative research (COREQ).

### Setting and participants

Sampling was done from five provinces (Tehran, Fars, Hormozgan, Hamadan, and Kerman) in Iran from April 2022 to mid-March 2023. The participants were managers and members of the EMS incident command team, EMS field experts, technicians, paramedics, and EMS telecommunicators with rich information who were selected using the purposive sampling method. Sampling continued until data saturation. The inclusion criteria were as follows: (1) having at least a bachelor's degree; (2) having experiences in MCIs context and being motivated to participate in the study; and (4) signing an informed consent form.

### Data collection

Data were collected through semi-structured interviews by the researcher (VS), using a pilot-tested interview guide. The interviews were conducted face-to-face, and their follow-up was mostly done using a telephone. The study outline was approved by three members of the research team. Some of the main interview questions were as follows:

1. In what ways is EMS more vulnerable during MCIs?
2. What measures are needed to be taken in EMS before MCIs?
3. What features should EMS systems have to respond to MCIs?
4. What elements affect EMS readiness in MCIs?

All the interviews were completed and recorded by the main researcher (VS). The interviews lasted about 30–90 min, and the participants responded to all the questions according to the research outline. Before the interview, the necessary arrangements were made with the participants. The interviews were conducted in a quiet and uninterrupted atmosphere. The researcher refrained from using negative, judgmental, and forgiving statements and attitudes. Data collection continued until saturation, and the researcher stopped sampling when he realized that no new data was obtained and that there were a lot of duplicated data.

### Data analysis

A qualitative method was used for data analysis. After each interview, the recordings were transcribed, and the main researcher (VS) used the content analysis method to analyze and summarize the data. The steps were: (1) familiarization: the text was read repeatedly to familiarize the subjects with the qualitative data; (2) coding: the open coding method was used to analyze the data line by line, and essential words and phrases (unit meaning) were recorded in the margins of the content; and (3) integration: each important meaning unit was described in a descriptive code, and codes with the same meaning were integrated [18]. MAXQDA was used for data organization and coding. After the analysis, two other researchers, who were familiar with the telephone records, performed a peer review to ensure the validity of the analysis results.

### Rigor

Long-term engagement with participants, member and peer checking, presentation of rich descriptions of data, and data analysis were used for rigor [19]. Besides long-term communication with the participants, the research team spent enough time on data collection and follow-up with the interviewees. Transcriptions along with coding and categories were shown to two qualitative researchers for peer check. Their confirmation was obtained after applying their comments. A brief report of the interviews and the extracted codes was given to the interviewees, who approved it to examine the members. There was also an attempt to consider the maximum diversity in sampling for transferability [20].

### Integrating the results of the systematic review and qualitative study

The integration of the results was done using joint display technique in the expert panel [21]. The members present in the panel consisted of 10 experts in the field of EMS and MCIs area who were selected purposefully. To integrate the findings of two qualitative stages and systematic review, we first entered a table with three columns into Word software. On the left side, the findings of the systematic review were entered, and on the right column, those of the qualitative study were entered. A column in the middle was considered empty to enter the integrated findings. Before entering the findings into the table, the research team did a content analysis on the findings of the systematic review, and new concepts were obtained from the data extracted from this stage. Two expert researchers in the field of EMS independently reviewed the results of this content analysis, and any disagreement was resolved. This content analysis was done because, firstly, the components of the systematic review were unique in terms of content and did not overlap with each other. Secondly, the components of the two stages should have a closer affinity for integration. It is worth mentioning that two researchers in the field of EMS and the people present in the panel were very familiar with the setting of EMS and this selection of people was done purposefully. After entering the final results of each step into the table, the panel members reviewed all the findings of both studies separately. Then, the components were read one by one from the left side by the researcher and the members were asked to determine the similarity of this finding with that of the right column by drawing a line through critical thinking. To reach a consensus at this stage, voting by raising hands was used. This process was done for all the findings in two consecutive panel sessions until the completion of the integration stage. Table 2 shows integration of components using the display joint method.

**Table 2:**
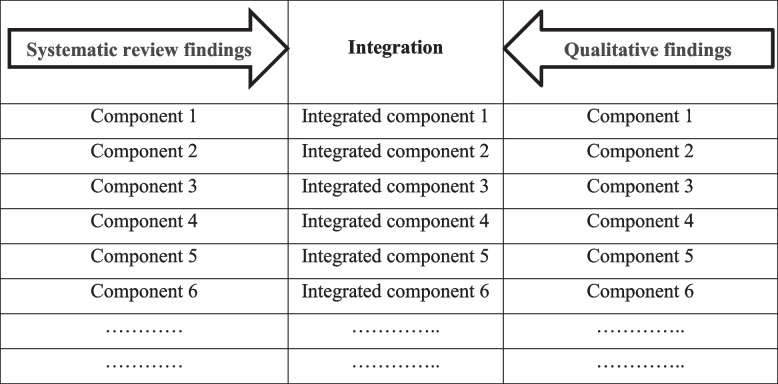
Display joint method for integrating the results

### Validation of the components using the Delphi technique

The Delphi technique was used to validate the integrated components. It is a systematic approach to elicit opinions from a group of experts on a specific topic [22]. Targeted sampling was used to select the experts who were working in EMS systems at this stage. The research population consisted of experts in the field of health in disasters and emergencies, emergency medicine specialists, and managers of the EMS system of Iran (Fars, Hormozgan, Tehran, Hamedan, and Kerman provinces). A questionnaire containing extracted components was created by the research team and sent to 29 participants who were selected purposefully. This questionnaire included 23 questions scored using a five-point Likert scale, and the scored ranged from completely agree (score 5); I agree (score 4); I have no opinion (score 3); I disagree (score 2), and completely disagree (score 1). In the questionnaire form, the purpose of this step was explained, and the consent form was signed by the participants. Out of 29 people, 22 completed the questionnaire. SPSS version 16 software was used for data analysis. Descriptive statistics were used to interpret the data. Mean and standard deviation indicators were used to describe the percentage of the participants' agreement on each component. In the first round, the components whose average score was less than 50% were eliminated. A percentage of agreement greater than 75% for each component means that it is accepted. The components whose percentage of agreement was between 50 and 75% were included in the second round, and if more than 75% agreement was obtained, they were approved [23].

### Model design

A panel of experts was used to design the model. This panel was held with the presence of 7 disaster context specialists and EMS experts who selected purposefully. For this purpose, the objectives of the research were first described to the experts. Then, the components obtained from the previous steps were explained to them so that they could express their opinions and ideas about the role and placement of the variables, and schematic of the model. Since in this model the cause-effect relationship of the variables was not considered, an attempt was made to design a simple and expressive model by considering the international standards. Therefore, to examine the models, we searched internationally recognized EMS agencies and systems such as the Federal Emergency Management Agency (FEMA), Pan American Health Organization (PAHO), and other related databases. After reviewing and discussing, a model that was close to our goals was selected finally. However, minor changes were made to this model to make it easier to understand. This model was introduced by Adam Tager. Adam Tager serves as the disaster preparedness program manager in the FEMA mission support executive office. In this role, he leads preparedness and response attempts on behalf of the associate administrator for mission support. Before this, he served as a field operations analyst and a consultant supporting FEMA and the Department of Defense. He has worked with national and state emergency management programs and has had roles in all-hazards event response and recovery, development and conduct of training exercises, and development and writing after-action reports [24]. Therefore, this model was selected, and after some changes was made in it, the schematic of the model was drawn.

## Results

### Findings of the systematic review

After searching the databases, 20,499 articles were identified; of them, 201 were in Direct Science, 1417 in PubMed, 16,192 in Scopus, 2677 in ProQuest, and 12 in the Cochrane Library. After screening and evaluating the quality of the articles, finally, 10 articles were included in the study. The components of the EMS preparedness in MCIs were identified after reviewing the articles. The 16 main components including education and training, skills and experience, relationships and psychological factors, management planning, constant improvement, coordination, manpower, equipment and ambulance, support processes, command and control, incident scene, triage, evacuation and gathering, treatment, communication, distribution, transport, and follow-up of the injured were extracted. These were extracted by the research team through summarizing the findings of the articles. The components were classified into four categories, including individual improvement, group improvement, resources, and operations through thematic analysis [16].

### Findings of the qualitative study

**Fig. 2:**
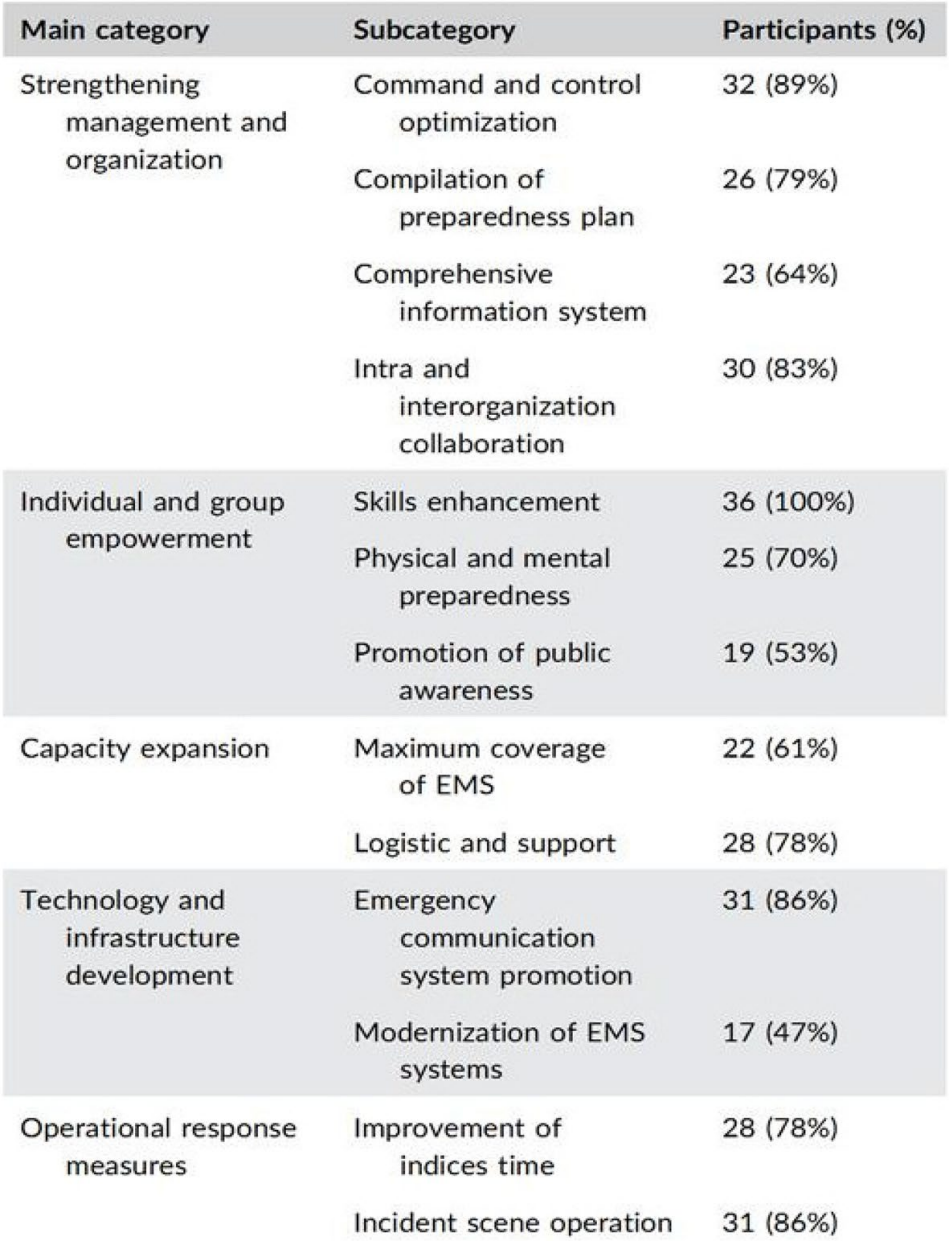
Findings of the qualitative stage (this printed table is the result of our article published in reference 20)

Thirty-six participants (24 men and 12 women) were included in the study. Thirteen components were extracted from the study and classified into five categories including strengthening management and organization, individual and group empowerment, capacity expansion, technology and infrastructure development, and operational response measures [20], as shown in Fig. 2.

### Findings related to the integration of the results of the systematic review and qualitative study

23 components were extracted from combining the results of the two stages of systematic review and qualitative study in the panel of experts. The integrated components are presented in Table 3.

**Table 3:** Findings related to the integration stage

N	Components	Systematic review	Qualitative study
1	Command and control	*	*
2	Internal and inter-organizational coordination	*	*
3	Education and training	*	*
4	Physical and mental preparedness	*	*
5	Resource management (mobilization and centralization of resources)	*	*
6	Increase capacity	*	*
7	Compilation of preparedness plan	*	*
8	Strengthening the infrastructure	*	*
9	Promotion of public awareness	*	*
10	field operations	*	*
11	Time indicators of emergency operations	*	*
12	Unified emergency contact number	*	*
13	Comprehensive system information	*	*
14	EOC integrity	*	*
15	Safety and security	*	*
16	Logistic and support	*	*
17	EMS modernization	*	*
18	Emergency communication	*	*
19	EMS maximum coverage	*	*
20	EMS decontamination teams in CBRNE	*	-
21	Ethical considerations	*	-
22	Common triage and treatment	*	-
23	Specialized tactical teams	*	*

*EOC* Emergency operation center

*CBRNE* Chemical, Biologic, Radioactive, nuclear, Explosive

### Findings of the Delphi phase

In the first round of Delphi, 22 out of 28 participants completed the questionnaire. In the second round of Delphi, out of 28 subjects who had received the questionnaires again, 22 experts filled out the questionnaires sent. Descriptive statistics showed that 22 subjects entered the Delphi stage, of whom 16 were men and 6 were women in the age range of 32–55 years and with a work experience of 5–25 years. The results of the statistical analysis showed that out of 23 components, only "Establishment of EMS decontamination teams in CBRNE" scored less than 2.5 (with an average score of 2.27, which means less than 50% of the acceptable score), so it was eliminated during the first round.

According to the results, the average of most of the components was more than 3.75 (more than 75% agreement), and only two components, "Common triage and treatment" and "Unified emergency contact number", had an average score of more than 2.5 and less than 3.75 (i.e. agreement between 50 and 75 percent) and were sent to the second round of Delphi. In the second round of Delphi, the two components mentioned were again given to 22 participants and the scores were analyzed. The results showed that the average agreement score of these components was more than 3.75 (i.e. more than 75% agreement).

After the completion of the Delphi phase, it was found that 22 out of the 23 components finally got the necessary points; thus, they were approved for review in the next phase which was related to the panel of experts. All the Delphi rounds and the final scores are shown in Table 4.

**Table 4:** Scores of the components in the rounds of Delphi

N	Components	First round of Delphi (Mean/Standard deviation)	Second round of Delphi (Mean and standard deviation)	Final score	Final result
1	Command and control	4.77 / 0.42	-	95.45	Accept
2	Internal and inter-organizational coordination	4.90 / 0.29	-	98.18	Accept
3	Education and training	4.95 / 0.21	-	99.00	Accept
4	Physical and mental preparedness	4.63 / 0.90	-	92.72	Accept
5	Resource management (mobilization and centralization of resources)	4.72 / 0.45	-	94.54	Accept
6	Increased capacity	4.86 / 0.35	-	97.27	Accept
7	Compilation of preparedness plan	4.86 / 0.35	-	97.27	Accept
8	Strengthening the infrastructure	4.86 / 0.35	-	97.27	Accept
9	Promotion of public awareness	4.63 / 0.49	-	92.72	Accept
10	field operations	4.90 / 0.29	-	98.18	Accept
11	Time indicators of emergency operations	4.81 / 0.39	-	96.36	Accept
12	Unified emergency contact number	2.63 / 0.58	3.81 / 0.85	76.36	Accept
13	Comprehensive system information	4.72 / 0.45	-	94.54	Accept
14	EOC integrity	4.59 / 0.50	-	91.81	Accept
15	Safety and security	4.72 / 0.55	-	94.54	Accept
16	Logistic and support	4.81 / 0.39	-	96.36	Accept
17	EMS modernization	4.77 / 0.42	-	95.45	Accept
18	Emergency communication	4.86 / 0.35	-	97.27	Accept
19	EMS maximum coverage	4.77 / 0.42	-	95.45	Accept
20	EMS decontamination teams in CBRNE	2.27 / 0.70	-	45.45	Reject
21	Ethical considerations	4.63 / 0.58	-	92.72	Accept
22	Common triage and treatment	2.50 / 0.74	3.77 / 0.75	75.45	Accept
23	Specialized tactical teams	4.63 / 0.58	-	92.72	Accept

### Model design

**Fig. 3:**
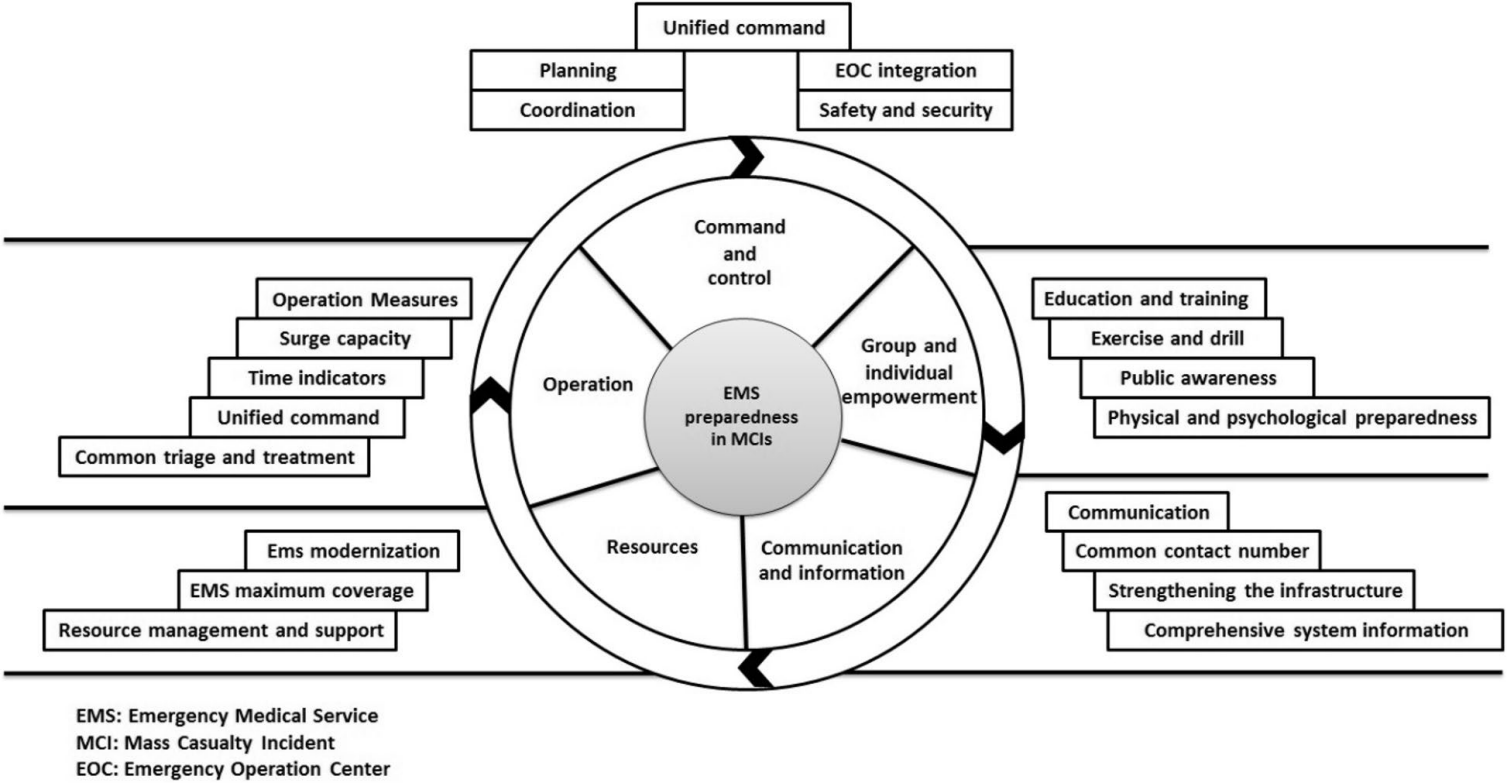
The model of EMS preparedness in MCIs

The design of the schematic was based on the model proposed by Adam Tager. According to the acceptability and validation of this model in the panel of experts, with some modifications in line with the study objectives, this model was selected and finally the schematic form of the model was designed. This pattern consists of classes that are placed in a cycle, while the main subject of the model is in the center, and the subclasses are placed outside the cycle in their partitions. After content analysis in the panel, all components were placed in 5 main categories including "Command", "Operations", "Resources", "Communications and Information", and "Group and Individual Empowerment". The final model of EMS preparedness in response to MCIs is designed in Fig. 3.

## Discussion

After several consecutive steps in this research, the components of EMS preparedness in MCIs which were the main objectives of the study were finally determined. One of the reasons why the research team decided to do a mixed-method work was the importance of the area. Maybe, if we had a single study approach to achieve the main goal, we could not confidently trust the obtained result. Therefore, the mixed method was adopted. Systematic review studies contain rich results, and we have made comprehensive findings in this phase. The findings of the systematic review included the experiences of EMS systems around the world in response to MCIs. Most of the results of this phase, according to the selected articles, were taken from the real experiences of EMS systems, and this was a turning point in the findings obtained in this research. Considering that this study was in a special context (the field of emergencies and disasters), it would be better to consider complementary methods to achieve the goals. Therefore, we decided to look for unwritten experiences in disaster context, in addition to the published documents. For this reason, we chose a qualitative approach to obtain the experiences of people who spend their lives in EMS systems. The purpose of conducting a qualitative study was to enrich the results in this mixed method. A qualitative study was conducted with in-depth semi-structured interviews in Iran, and the results obtained were mostly consistent with those of the systematic review phase. After passing through these two steps, we integrated the findings in a systematic way to obtain more comprehensive results. After several consecutive stages, 22 main components were finalized, which were drawn in a conceptual model in 5 main levels including command, operation, resources, communication and information, and group and individual empowerment for better understanding and implementation. Although the known components in this research have many similarities with the studies done, the differences mostly originate from regional and national structures and policies. One of the highlights of this study was the removal of the component of "EMS decontamination teams in CBRNE" in the first round of Delphi. Although this component was extracted from the systematic review [16], it was not accepted in the remaining stages, including the qualitative study [20], Delphi stage, and panel of experts. One of the reasons this issue arose in the qualitative study and expert panel was that EMS has complex tasks in triage, treatment, transfer, and other incident management processes [25], and decontamination is a sensitive operation that requires specialized logistics and equipment that is beyond the scope of most EMS systems. In most emergency systems, the duty of decontamination (according to the type of the risk) is the responsibility of the fire department, army, safety units of refineries and factories, and so on. However, this component must be accepted in some EMS systems due to the existence of resources, equipment, integration of rescue teams, and regional policies [26]. Due to the complexity of CBRNE incidents, regional policies are essential, and this component may not be accepted globally in an EMS setting. However, this issue depends on national and regional policies.

In addition, components such as a common emergency contact number, common triage and treatment system, and tactical teams that are used in many emergency systems still have pros and cons in some countries. However, the rest of the components, including the unified command, strengthening of communication, education and training, physical and psychological factors, and so on, are present in most EMS systems as the main indicators of readiness although there may be differences in how they are implemented. Nonetheless, in this research, the components of EMS preparedness in MCIs were explored in several stages so that a model with reliable indicators for planning and policymaking can be developed.

Various methods have been used in developing EMS preparedness and response models in MCIs. Among these, dynamic modeling methods, computer modeling, conceptual modeling, and modeling with cause-effect relationships can be mentioned.

A study by Lee aimed to simulate the distribution of emergency relief equipment for disaster response operations. In this study, the concern was that in the event of disasters such as storms, earthquakes, and terrorism, there is a need to distribute emergency relief equipment to the victims to protect their health and lives. The research team developed a modeling framework for disaster response in which the supply chain of relief supplies and distribution operations were simulated and analyzed to test the optimal transportation of relief supplies to various distribution points. The disaster response simulation model included the modeling of the relief resource supply chain and operations at the distribution point. The results showed that the model could evaluate a wide range of disaster scenarios, evaluate disaster response plans and policies, and identify better approaches for government agencies and first responders [27].

In another study, a simulated model of EMS services in MCIs was designed by Su in Taiwan. In this study, object-oriented simulation software was used to improve EMS care. In this research, a computer virtual simulated model was designed. The results showed that the most efficient part of this model in caring for the injured is when the integrated deployment of EMS is launched along with the increase of emergency networks and specialized life-saving protocols [28].

Another research was conducted by Pasupathy in Belgium to design a simulated model of casualty management in disasters and emergencies. In this study, a profile of the real injured was drawn by the elites of the pre-hospital field. These profiles were drawn in a medical emergency model where a single response was given by the system to real casualties. The medical emergency response model focused on emergency services operations including triage, evacuation, and medical procedures. Medical decisions such as whether to evacuate or treat at the scene were based on the victim’s breathing, heart rate, and motor response. Finally, a simulated model was designed that was related to road accidents and showed how much resources could affect the prognosis in these incidents [29].

In China, a model entitled EMS response dynamic model was designed in response to MCIs. This study was conducted to find out the EMS-MCI modeling in Shanghai, improving rescue efficiency in MCIs and providing a possible method for quick decision making in these incidents. This model was designed using the Vensim DSS program and intervention scenarios by adjusting the scales of accidents, ambulance allocation, emergency medical staff allocation and the efficiency of organization and command. The results showed that by increasing the number of ambulances and improving the efficiency of the organization and command, the mortality rate decreased significantly [30].

Tseng in China designed a theoretical model of EMS response in tunnel-related traffic incidents as a scenario-based computer simulation model. In this study, based on a theory, data related to the general characteristics and components of MCIs were collected in order to create a simulation model based on the method of emergency response plans. In this method, a disaster response simulation model was presented using realistic accidents taken from previous experiences. In this study, the main variables included EMS response components in MCIs, pre-hospital time indicators, the ability to save and preserve hospital life, and the level of organizational and command efficiency. This model is called causal curve diagram and included 5 main parameters with 102 variables, which were connected in the form of curvilinear flows and based on one-way or two-way relation. In addition, the subsystems of this model include 5 items: MCIs, hospital rescue and life preservation operations, organization and control, emergency center, and finally prognosis of the injured which were connected through input and output variables [31].

The results of this mixed method indicate that EMS systems need to strengthen specific components to increase their readiness in response to MCIs. The components extracted from this study were identified in depth with several approaches which were finally drawn in the form of a simple model. Due to the deep and scientific view in the methodology of this research and the extraction of validated findings, these results can be theoretically used in EMS planning and policies in the field of disasters. In addition, EMS systems and Partner organizations can use the results of this research in practice and personnel training. Due to the importance of the mixed method in achieving rich results in the field of disasters, the method of this study can be used more than single approaches. Also, the methodology and techniques of this research can be used as a pattern in the design of future studies in important areas such as disasters where it is necessary to achieve valid and vital results.

Due to the complexity of management and operational planning in most MCIs, it would be better to draw the preparedness plans in a simple and comprehensible way so that a quick and effective response can be delivered. In this research, the goal was to create a simple conceptual model that represents the main components of EMS preparedness in MCIs. The most important distinguishing feature of the model designed in this study was that simulation, computer and special software methods were not used as in the above-mentioned studies and the main attempt was to design a model to cover the important readiness indicators of EMS systems in response to MCIs so that they can be used easily. Although most of the components were identified and validated in this study, there may be other components that can be unique to states and regional policies. For example, some specific indicators were related to the regions with specific climatic and geographical conditions that overshadow the EMS response. However, in this research the components of EMS preparedness in MCIs were only introduced and presented in the form of a model, and the effectiveness of this model was not measured practically. Therefore, it is recommended that the effectiveness of this model in the management of MCIs in practical exercises, simulations and real incidents should be measured.

### Limitations

In the systematic review phase, one of the limitations was the lack of access to some electronic databases such as Web of science (WOS), for searching articles. Unfortunately, due to economic and political sanctions in Iran, some databases were not available at the time of the search and we had to ignore them. This issue worried us because we might have missed some studies. However, we tried to use other reliable electronic databases such as PubMed, Scopus, Science direct, ProQuest, and Cochrane.

## Conclusions

MCIs are complex conditions that can seriously overwhelm the EMS function. Improving EMS readiness in MCIs is multifactorial and influenced by regional and national conditions. Designing a model with different methods in improving EMS readiness in MCIs can have a significant impact on better understanding of the plans and policies in simulated environments and real incidents. The designed model can be used as a framework for implementing EMS management strategies in MCIs.

## Data Availability

The data are not publicly available due to privacy or ethical restrictions, but the data that support the findings of this study are available on reasonable request from the corresponding author.

## Abbreviations

MCI: Mass Casualty Incident
EMS: Emergency Medical Service
WHO: World Health Organization
EOC: Emergency Operation Center
CBRNE: Chemical, Biologic, Radioactive, Nuclear, Explosive
NIMS: National Incident Management System
ICS: Incident Command System
PRISMA: Preferred Research Items for Systematic review and Meta-Analysis
FEMA: Federal Emergency Management Agency
PAHO: Pan American Health Organization
COREQ: Consolidated criteria for reporting qualitative research
STROBE: Strengthening the Reporting of Observational Studies in Epidemiology
